# Structural and electronic properties of graphene nanoflakes on Au(111) and Ag(111)

**DOI:** 10.1038/srep23439

**Published:** 2016-03-22

**Authors:** Julia Tesch, Philipp Leicht, Felix Blumenschein, Luca Gragnaniello, Mikhail Fonin, Lukas Eugen Marsoner Steinkasserer, Beate Paulus, Elena Voloshina, Yuriy Dedkov

**Affiliations:** 1 Fachbereich Physik, Universität Konstanz, 78457 Konstanz, Germany; 2 Institut für Chemie und Biochemie, Freie Universität Berlin, 14195 Berlin, Germany; 3 Humboldt-Universität zu Berlin, Institut für Chemie, 10099 Berlin, Germany; 4 IHP, Im Technologiepark 25, 15236 Frankfurt (Oder), Germany

## Abstract

We investigate the electronic properties of graphene nanoflakes on Ag(111) and Au(111) surfaces by means of scanning tunneling microscopy and spectroscopy as well as density functional theory calculations. Quasiparticle interference mapping allows for the clear distinction of substrate-derived contributions in scattering and those originating from graphene nanoflakes. Our analysis shows that the parabolic dispersion of Au(111) and Ag(111) surface states remains unchanged with the band minimum shifted to higher energies for the regions of the metal surface covered by graphene, reflecting a rather weak interaction between graphene and the metal surface. The analysis of graphene-related scattering on single nanoflakes yields a linear dispersion relation *E*(*k*), with a slight *p*-doping for graphene/Au(111) and a larger *n*-doping for graphene/Ag(111). The obtained experimental data (doping level, band dispersions around *E*_*F*_, and Fermi velocity) are very well reproduced within DFT-D2/D3 approaches, which provide a detailed insight into the site-specific interaction between graphene and the underlying substrate.

Graphene, a flat monolayer of carbon atoms arranged in a honeycomb lattice, is of particular interest as a possible material for many electron- and spin-transport devices^1^,^2^. Recent progress in the utilization of metal surfaces for the synthesis of polycrystalline graphene layers, which might later be transferred onto a polymer support and used for the fabrication of, e.g., touch screens^3^,^4^, renewed the interest to the surface science studies of graphene/metal interfaces^5^,^6^,^7^,^8^. This makes a comprehensive study of graphene-metal-contacts inevitable, as the graphene-derived valence band states are highly susceptible to the overlap with the metal-derived states that can lead to drastic changes in the properties of graphene^9^,^10^. According to the present considerations, the energy spectrum of the carriers of graphene, which is in contact with a metal, is always strongly perturbed (doping, gap openings, hybridizations of the graphene- and metal-derived states) leading to the loss of the massless character of carriers around the Fermi energy (*E*_*F*_) and the Dirac point (*E*_*D*_). However, theoretical and experimental investigations allow a distinction between graphene which is *weakly* and *strongly* interacting with metals. In the first case [as an example, graphene on Au(111), Ag(111), Cu(111), Ir(111)]^11^,^12^,^13^,^14^,^15^,^16^,^17^ graphene can be either *n*- or *p*-doped with a linear dispersion around *E*_*D*_. In the latter case of graphene interacting *strongly* with a metal [examples are graphene on Ni(111), Rh(111), Ru(0001)]^14^,^18^,^19^,^20^,^21^,^22^, a very short graphene-metal distance, *n*-doping of the graphene layer as well as a strong band bending and hybridization, leading to a complete destruction of the linear dispersion of graphene at *E*_*D*_, are observed.

Additional interest in the graphene/metal systems is connected with the progress in the fabrication of graphene nano-objects, like nanoribbons (GNRs)^23^,^24^, nanoflakes (GNFs)^25^,^26^,^27^, quantum dots (GQDs)^28^,^29^,^30^, and nanojunctions^31^. Such objects of reduced dimensionality can demonstrate the strong modification of the energy spectrum of graphene charge carriers, like gap formations, appearance of spin-split edge states, mass renormalization or hybridization with the states of the metal, thus boosting the interest in well-defined graphene nano-objects on weakly-interacting metal substrates.

In the present work, the structural and electronic structure of graphene nanoflakes on noble metal surfaces, Au(111) and Ag(111), was studied by means of scanning tunneling microscopy and spectroscopy (STM and STS). These systems were formed via intercalation of a thick layer of noble metal in GNFs/Ir(111). We show that STS measurements and the corresponding Fast-Fourier-Transform (FFT) analysis allow to unambiguously identify the scattering features arising from the metallic substrate and from graphene. The experimentally obtained energy dispersions of the charge carriers for the metal surface state electrons and for charge carriers in graphene are analyzed relying on state-of-the-art density functional theory (DFT) calculations, providing detailed information about the graphene-metal interaction.

## Results and Discussion

### Structural properties of graphene on Au(111) and Ag(111)

**Figure 1:**
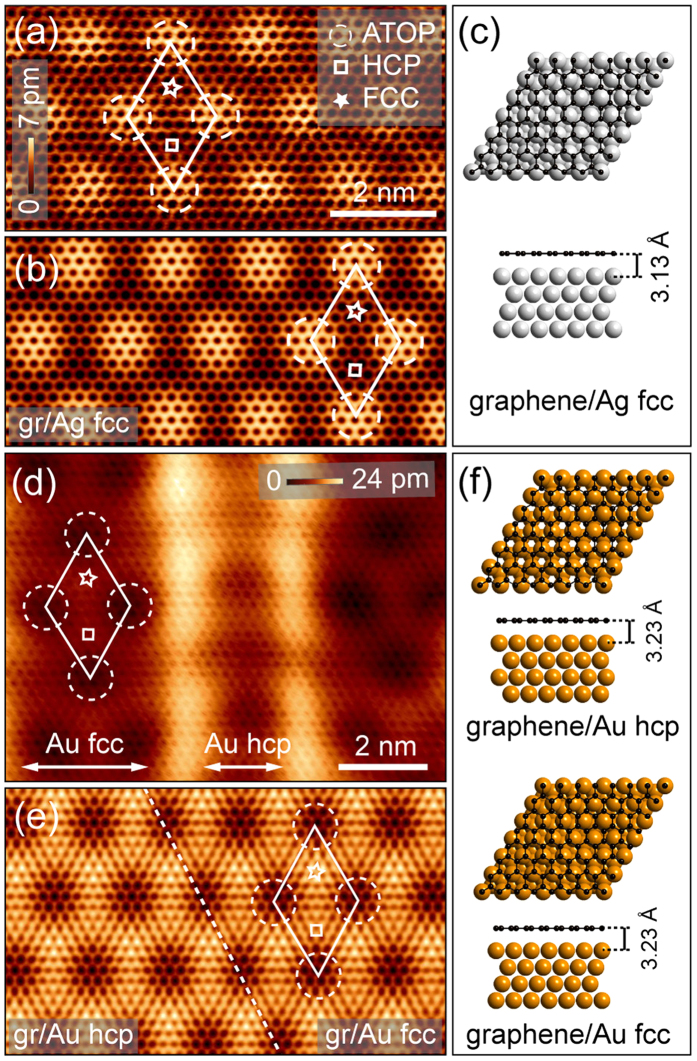
(**a**) Topographic image of graphene on Ag(111) including a depiction of the moiré unit cell. ATOP, HCP and FCC positions of the moiré unit cell are marked. (**b**) DFT simulated STM image of the (7 × 7)/(6 × 6) graphene/Ag(111) system. (**c**) Top and side view of graphene/Ag(111) structure. (**d**) Atomically resolved STM topography of graphene on Au(111). (**e**) Corresponding simulated STM images of graphene/Au(111) obtained from DFT simulations. (**f**) Top and side view of graphene on the hcp and fcc Au(111) surface. Imaging parameters: (**a**) *V* = 100 mV, *I* = 1.5 nA, *T* = 11 K; (**d**) *V* = 50 mV, *I* = 2.3 nA, *T* = 12.3 K.

Structural properties of graphene/Au(111) and graphene/Ag(111) were studied by means of STM at the atomic scale and compared with the results of DFT calculations. Figure 1 shows atomically resolved STM images of (a) graphene/Ag(111) and (d) graphene/Au(111) in comparison to the corresponding simulated STM images (b) and (e). Top and side view of the DFT optimized structures for graphene/Ag(111) and graphene/Au(111) are shown in (c) and (f). Moiré structures with the periodicity of 16.4 Å and 17.0 Å for Ag(111) and Au(111), respectively, were observed in STM due to the lattice mismatch between graphene and Metal(111) surface [15.8% for graphene/Ag(111) and 14.7% for graphene/Au(111)]. In Fig. 1 the so-called *R*0 structures are considered when the metal 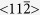 direction is parallel to the graphene 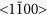 direction. Both experimental and calculated STM images clearly demonstrate all high-symmetry positions of the moiré supercell^9^ and these sites are marked by the respective symbols on all images (ATOP – dashed circle, HCP – square, FCC – star). Graphene on Ag(111) is imaged in the direct contrast, whereas graphene on Au(111) is imaged in the so-called *inverted* contrast for the low bias voltages used for the atomically-resolved imaging. In the latter case, the ATOP positions of the moiré structure are imaged as dark spots and other sites are brighter in the STM images. A similar effect was found also for the graphene/Ir(111) system and here this effect was assigned to the moiré-structure modulated interaction that leads to the formation of sites in the graphene moiré structure, where interface states are formed that are responsible for the observed STM imaging contrast^32^,^33^. In addition, graphene/Au(111) shows a herringbone reconstruction with a corrugation of 17 ± 1 pm of the graphene-covered Au(111) surface which remains intact upon graphene adsorption. The switching of the Au surface stacking across Shockley partial dislocation lines from Au fcc to Au hcp areas brings about a permutation in high symmetry moiré sites leading to a discontinuous moiré superstructure across the herringbone reconstruction lines. However, the graphene/Au(111) moiré structure itself does not depend on the metal stacking underneath (fcc or hcp), which can be assigned to the extremely weak interaction at the interface in this system.

DFT calculations yield an almost flat graphene layer in both systems. The corresponding binding energies (in meV/atom) and graphene-metal distances (in Å) calculated within DFT-D2 or DFT-D3 approaches are presented in Table 1. Extracted graphene corrugations, calculated as (*z*_max,C_ − *z*_min,C_), are 9.9 pm and 10.8 pm for graphene/Au(111) and graphene/Ag(111), respectively. These values are very close to the ones of the moiré corrugation of 4 ± 1 pm and 6 ± 1pm, respectively, obtained from STM experiments. In order to take into account the presence of the herringbone reconstruction, two absorption configurations - graphene/Au fcc and graphene/Au hcp were considered in the DFT calculations [Fig. 1(e,f)]. Both configurations yielded almost identical values for the graphene-metal distance and adsorption energy. Thus we do not expect any strong influence of the herringbone reconstruction on the electronic properties of graphene. All presented results refer to the graphene/Au fcc adsorption configuration.

**Figure 2:**
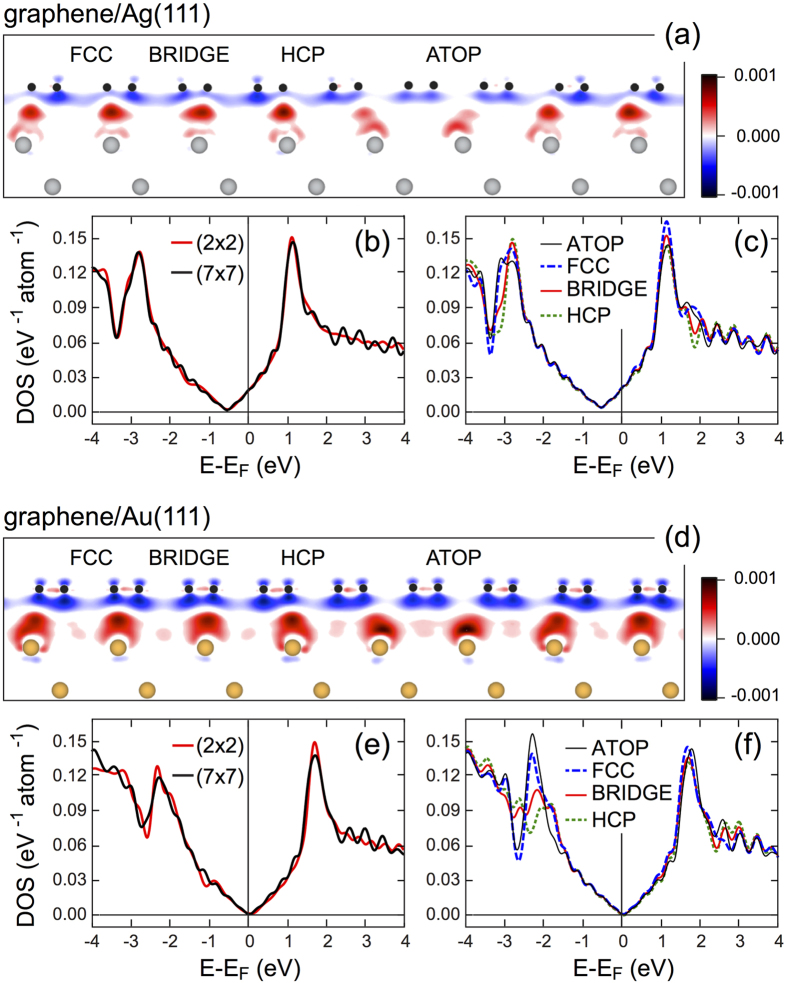
Difference electron density plotted in units of *e*/Å^3^ for graphene/Ag(111) (**a**) and graphene/Au(111) (**d**). (**b,e**) DOSs for graphene/Ag(111) and graphene/Au(111), respectively, calculated for two graphene supercells on metals, (2 × 2) and (7 × 7). (**c,f**) Graphene moiré lattice site projected DOSs for graphene/Ag(111) and graphene/Au(111), respectively, calculated for the (7 × 7) graphene supercell on metal surfaces.

Figure 2(a,d) shows side views of the graphene/Ag(111) and graphene/Au(111) structures obtained after geometry optimization overlaid with the corresponding difference electron density calculated as Δ*ρ*(r) = *ρ*_Gr/M_(r) − *ρ*_M_(r) − *ρ*_Gr_(r) and plotted in units of *e*/Å^3^, where M stands for metal. As can be deduced from these data, the charge distribution at these graphene/metal interfaces is different reflecting the respective interaction strength between graphene and metal surface. For the graphene/Au(111) system, the Δ*ρ* distribution is very similar to the one for gr/Ir(111)^17^,^32^, where the charge accumulation (depletion) on metal (graphene) is observed. In this system, graphene is weakly bonded to the metallic Au(111) substrate and it is *p*-doped with the Dirac point located at *E*_*D*_ = 0.05(0.17) eV above *E*_*F*_ as obtained from DFT-D2 (DFT-D3) calculations [Table 1 and Fig. 2(e,f)]. The situation for graphene on Ag(111) is opposite to that of graphene/Au(111) and the resulting charge distribution is similar to the one for the graphene/Cu/Ir(111) system^16^ with the charge depletion (accumulation) on metal (graphene). Graphene becomes *n*-doped after its adsorption on Ag(111) with *E*_*D*_ = −0.53(−0.41) eV, from DFT-D2 (DFT-D3) [Table 1 and Fig. 2(b,c)]. In this case also the *bond-like* states are formed at the HCP and FCC positions of the graphene/Ag(111) moiré structure similar to the graphene/Cu interface. The comparison of the carbon site projected partial density of states (PDOS) shows that the difference between the different high-symmetry positions for both systems is negligible [Fig. 2(c,f)]. The main difference between PDOSs can be found in the energy range 3–4 eV below the Fermi energy, where Au 5*d* and Ag 4*d* states are localized and overlap in the energy space with graphene *π* states. However, this effect does not influence the observed energy dispersion of graphene *π* states around the Fermi energy studied in this paper.

### Dispersions of metal-derived surface states

**Figure 3:**
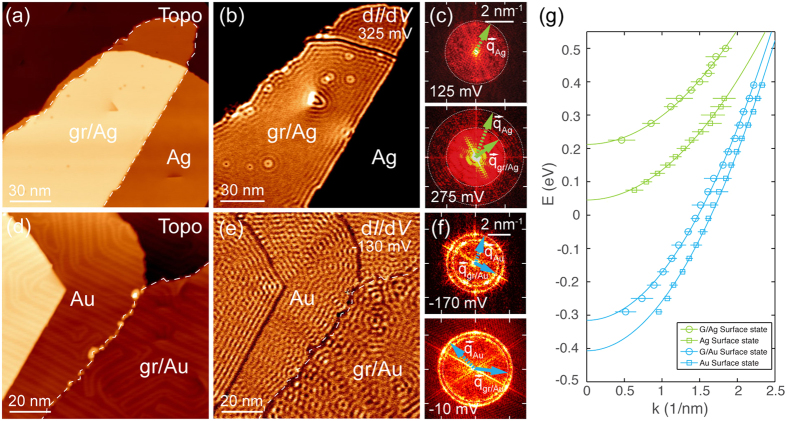
(**a**) Topography of a GNF on Ag(111) with few defects and dislocations. (**b**) d*I*/d*V* mapping on graphene and Ag surface exhibiting Friedel oscillations of two distinct wavelengths at defects and edges. (**c**) FFT of d*I*/d*V* maps obtained at *V* = 125 mV and *V* = 275 mV. Two circles corresponding to the standing waves on graphene and pure Ag can be identified. (**d**) Topography of a GNF on Au(111). (**e**) d*I*/d*V* map of the same region, also displaying two distinct oscillation wavelengths for graphene and pure Au. (**f**) FFT of d*I*/d*V* maps obtained at *V* = −170 mV and *V* = −10 mV. The corresponding surface state scattering vectors for graphene covered and pure Au are marked. (**g**) Dispersion relation *E*(*k*) of Ag and Au surface states on clean and graphene covered metal surfaces determined from the scattering vectors 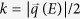. Measurements on Ag and Au were performed at 10 K and 8.3 K, respectively. Tunnelling parameters: (**a**) *V* = 500 mV, *I* = 200 pA; (**b**) *V* = 325 mV, *I* = 750 pA, *V*_mod_ = 5 mV, *f*_mod_ = 789.4 Hz; (**d**) *V* = −450 mV, *I* = 500 pA; (**e**) *V* = −130 mV, *I* = 600 pA, *V*_mod_ = 4 mV, *f*_mod_ = 665.0 Hz.

Quasiparticle interference in the proximity of defects leads to standing wave patterns in the topography [Fig. 3(a,d)] and even more so in the d*I*/d*V* images [Fig. 3(b,e)] on both graphene covered and non-covered regions of the metal surface. The observed spatial modulation of the local density of states (LDOS) arises from the backscattering of the respective Shockley surface state electrons of Au(111) and Ag(111) and their wavelengths vary strongly with the applied tunneling voltage [Fig. 3(b,e)]. Such d*I*/d*V* maps can be used for the FFT analysis^34^,^35^ in order to extract the characteristic scattering vectors. Thus the dispersion of the surface state *E*(*k*) can be obtained upon measuring wave vectors at different energies. In the case of the studied noble metals, the backscattering process within the ring-like constant energy contour of the parabolic surface state centered at the Γ-point leads to a circle around 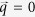 in the FFT images. The momentum *k* of the surface state electrons can further be obtained by using the relation *q* = 2*k*, with *q* being the radius of the scattering circle.

Figure 3(b,e) shows the experimentally obtained STM images with clearly visible standing wave patterns both on bare and graphene-covered noble metal surfaces. The corresponding dispersions of the surface states of Au(111) and Ag(111) obtained from the areas shown in the STM images for both clean and graphene covered surface are presented in Fig. 3(g). These dispersion relations *E*(*k*) for clean metal surfaces are parabolic as expected for the surface states and are in good agreement with previously published ARPES and STS data for Au(111) and Ag(111)^36^,^37^,^38^,^39^,^40^,^41^. The observed energy shift for the surface state band minima compared to the values reported for single crystals^42^,^43^ is attributed to the strain in the noble metal thin films^44^,^45^ and is subject to slight variations across the sample. For the regions of metal surfaces covered by graphene, the energy dispersions for the surface states display a similar parabolic dependence, but with the band minima shifted further upwards in energy with respect to the ones for the clean surfaces. This effect is explained by the stronger localization of the surface state wave function upon physisorption of a graphene layer on the metallic substrate. Such localization leads to the increased Pauli repulsion for these states and the corresponding increase of the energy of the surface state. Similar effects were also observed for the adsorption of atomic and molecular species^46^,^47^ as well as layered materials, i.e. *h*-BN^48^ or graphene^29^,^49^, on noble metal surfaces. Possible hybridization effects between metal *d* and graphene *π* states discussed before may, however, lead to a slightly shorter distance between graphene and metal, compared to the distance if only van der Waals interaction is considered. Such a reduction of the distance between graphene and noble metal will then lead to an even stronger localization of the surface state wave function, giving a small correction to the position of the band minimum. A quadratic fit of the obtained data points allows to obtain the position of the band minimum as well as the effective mass of charge carriers for the pure Au(111) and Ag(111) surfaces and for graphene covered Au(111) and Ag(111) as summarized in Table 2. As can be concluded from the behavior of the surface state electrons, which does not change substantially upon the presence of the graphene layer, the interaction between graphene and the Au(111) or Ag(111) surfaces is rather weak.

### Dispersions of graphene-derived states

**Figure 4:**
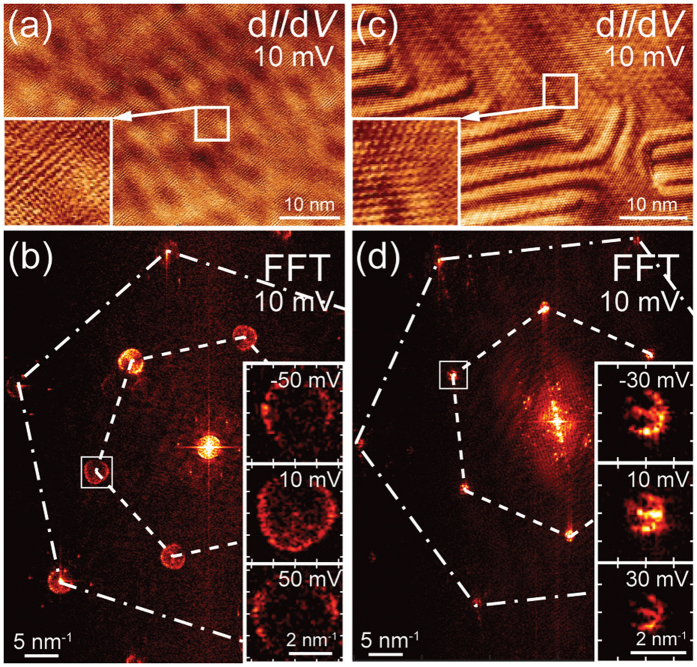
(**a**) Atomically resolved d*I*/d*V* map of graphene/Ag(111) with well visible LDOS modulations due to scattering. (**b**) Corresponding FFT of the d*I*/d*V* map displaying atomic spots, intra- and intervalley scattering. A zoom on the marked trigonally warped intervalley contour is shown for maps recorded at different energies, thus highlighting the change in intervalley radius. (**c**) Atomically resolved d*I*/d*V* mapping area for graphene/Au(111). (**d**) Corresponding FFT of mapping shown in (**c**). The zoom shows the ring-like intervalley contour, as the band structure can still be approximated by a cone at these energies. All measurements were performed at 10 K. Tunnelling parameters: (**a**) *V* = 10 mV, *I* = 800 pA, *V*_*mod*_ = 3 mV, *f*_*mod*_ = 789.4 Hz; (**b**) *V* = 10 mV, *I* = 1 nA, *V*_*mod*_ = 3 mV, *f*_*mod*_ = 672.0 Hz.

Along with the scattering circles of the Shockley surface state, additional features arising from scattering solely within the graphene flake are observed within the FFTs. These features can be assigned to two specific backscattering processes: scattering between two neighbouring Dirac cones (intervalley) and scattering within a single Dirac cone (intravalley)^25^,^34^,^35^,^50^. In the FFT images, intravalley ring-like structures appear at 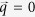 and around the atomic spots, while intervalley scattering rings are found at the 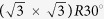 positions as can be seen in Fig. 4. It should be pointed out that in infinite perfect graphene layers the intravalley scattering is suppressed due to the conservation of pseudospin^34^,^35^, however, the lateral constrictions such as edges and steps of the investigated flakes as well as present defects may relax this requirement.

In the case of graphene/Au(111), the Au surface state scattering circle is still rather pronounced in the FT-LDOS within our measurement range of the electronic dispersion relation of graphene, whereas for graphene/Ag(111) the surface state is shifted towards the unoccupied states, therefore not being visible in the mapping energy range. The opening of a band gap in graphene at the Dirac point as observed in ARPES measurements^15^ lies outside our measurement range and can hence not be investigated further, as we observe enough scattering intensity only within the energy window of ±130 meV around *E*_*F*_.

**Figure 5:**
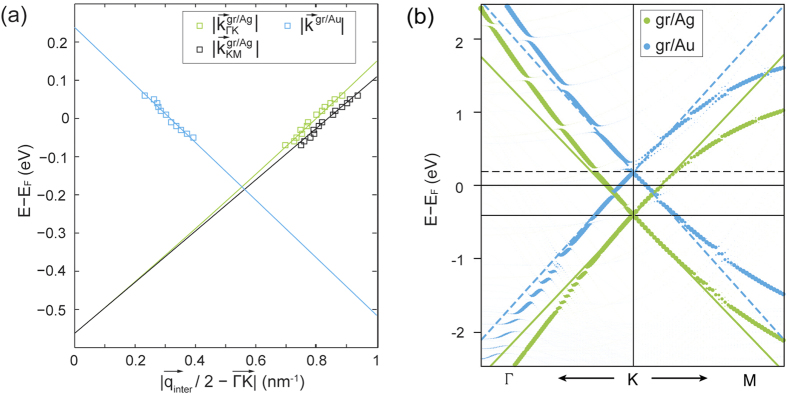
(**a**) Measured dispersion relation of graphene on Ag(111) and Au(111) obtained from the scattering analysis (open squares). Linear fits to the experimental data are used to extrapolate the position of *E*_*D*_ (solid lines). (**b**) Calculated energy band dispersions of gr/Ag(111) and gr/Au(111) for the (2 × 2) structures.

While the scattering features in the FFTs of graphene on Au(111) appear to be almost circular, the corresponding features on Ag(111) show a trigonal warping indicating a larger energy shift of the Dirac point with respect to the Fermi energy. This effect leads to an enlargement of the structures visible within the FFT, thus increasing evaluation precision, but also yielding variation of the Fermi velocity *v*_*F*_ depending on the direction in 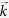-space. Plotting the measured scattering vectors versus the energy, the electronic dispersion relation for graphene on both noble metals can be traced from occupied to unoccupied states [Fig. 5(a)]. The experimentally obtained values of *E*_*D*_ and *v*_*F*_ extracted from the plotted dispersions are compiled in Table 3 together with the theoretical values obtained from the fit of the calculated band dispersions shown in Fig. 5(b) for both systems. In the case of graphene/Au(111), the energetic position of the Dirac point extrapolated from the experimental data is in agreement with previous results^51^ and fits better to the theoretical result obtained with PBE-D3. For graphene/Ag(111) both functionals yield a fairly reasonable agreement in terms of the position of the Dirac point. However, the PBE-D2 method delivers a value which is slightly closer to the experimentally determined one, thus being more appropriate for the graphene/Ag(111) system. The possible discrepancies between experimental and theoretical values may be attributed to a slight over-binding in the DFT-D2(D3) model, leading to a different graphene-metal distance. Graphene-metal distances of 3.13 Å (3.31 Å) and 3.23 Å (3.36 Å) have been determined for graphene/Ag(111) and graphene/Au(111), respectively, within the PBE-D2 (PBE-D3) approaches. As this length plays a crucial role in estimating the doping level, the obtained DOSs and band structures may be reproduced following further adjustment of this parameter.

## Conclusion

Graphene nanoflakes have been produced and investigated on Au(111) and Ag(111) in order to obtain information about their structural and electronic properties. Quasiparticle interference mappings on both pure and graphene covered Au and Ag have revealed scattering due to the metals’ surface state, which underneath graphene has been shifted towards lower binding energies. For both substrates, we find that the presence of graphene does not influence the behaviour of the surface state electrons. The possibility to observe metal-related and graphene-related scattering features allows us to trace each sample’s electronic dispersion relation separately for graphene and metal. Graphene/Au(111) exhibits a *p*-doping of 0.24 ± 0.07 eV, whereas graphene/Ag(111) shows a much larger *n*-doping of −0.56 ± 0.08 eV, hence already displaying trigonal warping at the Fermi energy. Despite the doping, the interaction between graphene and the chosen noble metal substrates is weak, since no additional band bending occurs within the measured energy range and the flakes appear to be quasi-freestanding. The obtained experimental data on the electronic structure of the flakes close to *E*_*F*_ are compared with the results obtained within DFT-D2/D3 approaches and good agreement between all data is found. However, for graphene/Au(111) the experimental data fits better to the DFT-D3 method, whereas DFT-D2 delivers a better result for the graphene/Ag(111) system.

## Methods

### Sample Preparation and STM Experiments

GNFs were fabricated on Au(111) and Ag(111) using the method described elsewhere^25^. In brief, graphene flakes on Ir(111) were prepared by temperature programmed growth^52^, subsequently 5 nm Au or 7.5 nm Ag were evaporated onto the as prepared flakes and intercalated in a post-annealing step at temperature of 720 K, yielding GNFs on well-ordered Au(111) or Ag(111) surfaces.

STM and STS measurements were performed at low temperatures in an *Omicron* Cryogenic STM under ultra-high vacuum (UHV) conditions (<5 ⋅ 10^−11^ mbar). Polycrystalline tungsten tips flash-annealed in UHV were used for all STM/STS measurements. The sign of the bias voltage corresponds to the potential applied to the sample. Differential conductance (d*I*/d*V*) maps were recorded by means of standard lock-in technique, using the modulation voltages (root-mean-square, rms) and frequencies given in the figure captions.

### DFT Calculations

DFT calculations were performed using the Vienna Ab initio Simulation Package (VASP)^53^ within the projector augmented wave method (PAW)^54^ with a plane wave basis set and the generalized gradient approximation as parameterized by Perdew *et al.*^55^. The long-range van der Waals interactions were accounted for by means of the DFT-D2 or DFT-D3 approaches^56^,^57^. Two types of models were considered for the gr/Ag(111) and gr/Au(111) systems: (i) a (7 × 7) graphene layer on top of a (6 × 6) Metal(111) surface and (ii) a (2 × 2) graphene supercell on a 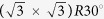 Metal(111) surface cell. Slabs of 5 and 15 metal layers with graphene on top were used in model (i) and (ii), respectively. For the geometry optimization the top two metal layers as well as the graphene layer were allowed to relax along the surface-normal until forces on the relaxed atoms along the surface-normal were lower than 0.01 eV/Å (model-i) and lower than 0.005 eV/Å (model-ii). For all cases a vacuum spacing of 14 Å was used in order to avoid unphysical interaction between periodic images of the slab. In all calculations a plane-wave energy cut-off of 400 eV was used. For the structure optimization a shifted Monkhorst-Pack *k*-space sampling of (4 × 4 × 1) (model-i) and (28 × 28 × 1) (model-ii) was used, where the Γ-point was explicitly included. After structure relaxation successive single-point calculations using denser (7 × 7 × 1) (model-i) and (39 × 39 × 1) (model-ii) *k*-grids were carried out in order to determine the density of states as well as binding energies and simulate STM images via the Tersoff-Hamann approximation^58^.

## Additional Information

**How to cite this article**: Tesch, J. *et al.* Structural and electronic properties of graphene nanoflakes on Au(111) and Ag(111). *Sci. Rep.* **6**, 23439; doi: 10.1038/srep23439 (2016).

## Figures and Tables

**Table 1:** Calculated adsorption energies (*E*_ads_, in meV/C-atom), equilibrium distances between graphene and metal (*d*_gr/met_, in Å) and position of Dirac point (*E*_*D*_, in eV) in graphene/Ag(111) and graphene/Au(111) as obtained with PBE-D2.

	graphene/Ag(111)		graphene/Au(111)	
	(2 × 2)	(7 × 7)	(2 × 2)	(7 × 7)
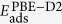 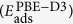	−94.90 (−74.34)	−94.24 (−73.28)	−114.18 (−78.24)	−113.71 (−77.84)
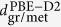 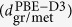	3.13 (3.31)	3.12 (3.29)	3.23 (3.36)	3.22 (3.37)
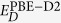 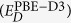	−0.53 (−0.41)		+0.05 (+0.17)	

Two supercell sizes are considered. In addition, the corresponding PBE-D3 results are given in parenthesis.

**Table 2:** Binding energies *E*_0_ and normalized effective mass *m* ^*^ of Au(111) and Ag(111) with and without graphene coverage.

	*E*_0_ (eV)	m^*^/*m*_0_
Au(111)	−0.41 ± 0.02	0.26 ± 0.02
graphene/Au(111)	−0.32 ± 0.02	0.27 ± 0.02
Ag(111)	+0.04 ± 0.02	0.39 ± 0.03
graphene/Ag(111)	+0.22 ± 0.01	0.40 ± 0.02

**Table 3:** Dirac point *E*_*D*_ and Fermi velocity *v*_*F*_ of graphene on Au(111) and Ag(111).

	*E*_*D*_ (eV)	*v*_*F*_ (m/s)
graphene/Au(111)^exp^	+0.24 ± 0.07	(1.2 ± 0.2) ⋅ 10^6^
graphene/Ag(111)^exp^	−0.56 ± 0.08	(1.0 ± 0.2) ⋅ 10^6^
graphene/Au(111)^DFT-D2^	+0.05	0.8 ⋅ 10^6^
graphene/Ag(111)^DFT-D2^	−0.54	0.8 ⋅ 10^6^
graphene/Au(111)^DFT-D3^	+0.19	0.8 ⋅ 10^6^
graphene/Ag(111)^DFT-D3^	−0.41	0.8 ⋅ 10^6^
